# Management of Mandibular Fractures at a High-Volume Educational Center—A Retrospective Study

**DOI:** 10.3390/jcm14186467

**Published:** 2025-09-13

**Authors:** Helya Hashemi, Yousuf Qundos, Payam Farzad

**Affiliations:** Department of Oral and Maxillofacial Surgery and Jaw Orthopedics, Karolinska University Hospital, Gävlegatan 55 NA2, SE-17176 Stockholm, Sweden; helya.s.hashemi@gmail.com (H.H.);

**Keywords:** mandibular fracture, mandibular trauma, malocclusion

## Abstract

**Background:** Mandibular fractures are often treated with open reduction and internal fixation in order to restore function and anatomy. This study analyzes postoperative complications and outcomes over one year at a high-volume teaching hospital, focusing on fracture types, treatment methods, and the impact of providers’ experience. **Methods:** This retrospective study included patients 12 years of age or older with mandibular fractures resulting from trauma during a 1-year period, January–December 2022 in a level 1 trauma center. Medical records were reviewed, and patient data was collected. Patients were categorized into 3 groups: Group 1 (surgical treatment), Group 2 (closed treatment, i.e., dental splints, arch bars/eyelets), and Group 3 (observation/soft diet). The results were tabulated, and standard descriptive statistics were used. **Results:** 141 patients with 223 mandibular fractures met inclusion criteria. Throughout all groups, 18 surgically treated patients (12.7%) and one patient treated with arch bars (0.07%) required additional unintended surgical procedure such as plate removal with/without re-plating, or orthognathic surgery for occlusal correction. **Conclusions:** The complication rates in this cohort align with the existing literature, though variations may origin from limited sample size, short follow-up and patient comorbidities. The involvement of less experienced surgeons during on-call hours most likely contributed to outcome variability. Despite challenges, most patients had favorable outcomes.

## 1. Introduction

Mandibular fractures are among the most common facial fractures, typically ranking first or second after nasal fractures. They occur more frequently in men aged 18 to 24 years, with assaults being a leading cause [1,2]. General goals of treating mandibular fractures are to re-establish the patient’s pre-traumatic occlusion, restore the bony anatomy of the mandible, and ensure adequate function. Currently, most mandibular fractures are effectively managed with open reduction and internal fixation (ORIF). This approach eliminates the need for postoperative intermaxillary fixation (IMF), shortens recovery time, improves functional outcomes, and ensures patient comfort [3].

Postoperative complications can occur in up to 15% of cases when treating mandibular fractures [4]. The mandibular angle has the highest overall complication rate (19%), while the body of the mandible is the most commonly affected site for nonunion following fracture repair in this anatomical region (3.2%) [5].

The most common complications include infection, osteomyelitis, malunion, nonunion, wound dehiscence, and hardware failure. Postoperative infections are considered the most common complication and, in the acute phase, are often related to compromised soft tissue and infected or fractured teeth left in the fracture line. Other contributing factors include poor oral hygiene, alcoholism, substance abuse, noncompliance with post-treatment care, poor reduction and inadequate fixation, as well as the severity and complexity of the fracture [4].

Several factors may influence complication rates, e.g., use of antibiotics, removal of tooth in the line of fracture, timing of treatment, surgical approach, fixation method, patient-related factors, and surgeon experience [6].

Studying the management of mandibular fractures in real-world clinical settings—especially within teaching hospitals where case complexity and provider experience can vary widely—offers valuable insights into clinical practice, complication rates, patient outcomes, and the overall quality of care. It also presents an opportunity to examine the impact of resident involvement and differing levels of experience on clinical outcomes, a topic that remains underrepresented in the current literature.

The objective of this retrospective study is to evaluate the postoperative complication rates associated with the management of mandibular fractures at a high-volume educational center. Specifically, it aims to assess how complication rates vary by fracture site and treatment method, and to compare these outcomes with those reported in the existing literature. It is hypothesized that the clinical outcomes in this cohort—particularly the rates and types of postoperative complications—will differ from those previously reported, reflecting the unique complexities and provider experience levels in a teaching hospital setting. The findings are intended to inform future treatment protocols and enhance patient care in comparable clinical environments.

## 2. Patients and Methods

Study Design

This study employed a retrospective observational design, analyzing clinical records of patients diagnosed with mandibular fractures over a one-year period.

Patient Population

Patients with mandibular fractures who presented to the Department of Oral & Maxillofacial Surgery and Jaw Orthopedics at Karolinska University Hospital, Sweden, between first of January throughout 31 December 2022 were included in this study.

Ethical Considerations

Written or verbal consent was obtained from all participants. The study was conducted in accordance with the Declaration of Helsinki and was approved by the Regional Ethics Review Board in Stockholm (registration number 2021-05691-01, date 15 February 2022).

Inclusion criteria

(1) Mandibular fracture resulting from trauma; (2) patients over the age of 12 with a permanent dentition; (3) adequate follow up (at least one follow-up appointment after surgery or initial consultation); (4) fracture confirmed by CT scan.

Exclusion criteria

(1) Patients with mixed dentition; (2) pathological fractures due to osteomyelitis, osteoradionecrosis, cysts or neoplasms; (3) incomplete medical records and/or inadequate follow up.

Clinical Feature Record

Medical records were retrieved by a medical secretary and subsequently reviewed by the authors (HH and YQ). The data collected from patient charts included: age, gender, comorbidities, cause of fracture, anatomical location of the fracture, involvement of teeth in the fracture line, time from injury to consultation/treatment, type of treatment, i.e., ORIF or closed, antibiotic regimen, occlusal status at the last visit, nerve impairment in relation to the inferior alveolar nerve (IAN) and/or the facial nerve (FN), and the occurrence and management of any complications. Patient allocation to treatment groups was based on clinical assessment, considering factors such as fracture type, displacement, occlusal stability, patient’s compliance and functional impairment. While institutional guidelines were followed, surgeon’s judgment influenced decisions in borderline cases. Although the degree of resident involvement was not specified in each case, residents are generally involved in both the surgical procedures and follow-up care.

Primary outcome measures included postoperative complications requiring additional surgical intervention, infection rates, and incidence of malocclusion. Secondary outcome measures involved fracture characteristics, treatment modality and approach, surgical timing, neurological outcomes, hospital stay duration, and the need for dental rehabilitation.

Statistical Analysis

The results were tabulated, and standard descriptive statistics were used. Categorical variables are presented as counts and percentages, while numerical variables are summarized using mean values. Demographic details of the enrolled patients are presented in Table 1.

## 3. Results

**Figure 1:**
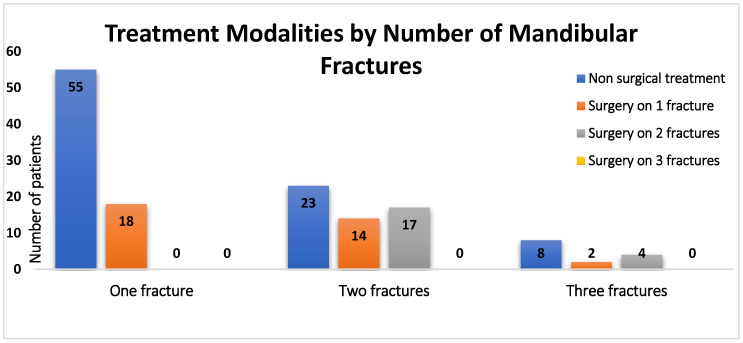
Patients and fracture incidence.

A total of 141 patients with 223 mandibular fractures were included in this study. The average age of the cohort was 30.2 years (range 14 to 100 years). The patients were predominantly male (*n* = 91, 64.5%). Main cause of injury was accidental falls (*n* = 40, 28.4%), followed by assault (*n* = 31, 22%), bicycle/electric motor accidents (*n* = 25, 17.8%), and syncope (*n* = 18, 12.7%). Other causes included sports accidents, epileptic seizures, gunshot wounds, and intoxication-related spasms (*n* = 27, 19.1%). A total of 73 patients had one single fracture, 54 patients had two, and 14 patients exhibited three fractures (Figure 1). Patients were categorized into three groups: Group 1 (surgical treatment), Group 2 (closed treatment, i.e., arch bars/eyelets/dental splints), and Group 3 (observation/soft diet).

Group 1: 55 patients with 99 fractures underwent surgical treatment for 78 fractures (Table 2). No single patient required surgical treatment for all three fractures. The most frequently surgically treated fracture was symphyseal (*n* = 27, 34.6%) followed by angle (*n* = 23, 29.5%), subcondylar (*n* = 15, 19.2%) and body (*n* = 13, 16.7%). The most common combination of fractures was in the condyle and symphysis (*n* = 9, 16.3%), followed by the angle and symphysis (*n* = 7, 12.7%).

All patients were prescribed antibiotics postoperatively for 7–10 days. In total, 23 patients (41.8%) developed a surgical site infection (SSI) (i.e., pus or granulation tissue at the surgical incision), necessitating extended antibiotic treatment. The majority of infections occurred within the first two weeks following surgery (74%). In 11 patients (48%), at least one fracture was successfully treated with antibiotics. Removal of the osteosynthesis was required in 12 patients (52%), and in 5 patients the removed osteosynthesis had to be replaced with new hardware (See details in Table 3). In five additional patients, bone plates were removed due to radiographic evidence of lysis and patient discomfort.

In summary, a total of 17 patients required a second intervention for plate removal, of whom 5 required new hardware following the initial surgery. 

In total, 33 patients (60%) were operated on during regular office hours, while the remaining 22 patients (40%) underwent surgery during on-call hours. The majority of patients (38/55, 70%) required one day or less of inpatient care, while the remaining 17 patients (30%) needed more than one day of inpatient care.

In total 55 fractures were approached trans-orally while 23 fractures required a trans-cutaneous incision. Thirty-seven patients (67%) reported neurosensory alteration of the IAN (mean follow-up period of 6.1 months). However, only 34% could be attributed to surgical intervention disregarding potential trauma-induced injuries. Eight patients (34.8%) exhibited visible motor deficits in the facial muscles following retromandibular (*n* = 3, 13%) and submandibular (*n* = 5, 21.7%) incisions (mean follow-up of 9 months).

In total, 58 teeth were involved in the fracture line. Furthermore, 23 were extracted during surgery and 2 teeth after surgery or during re-operation for non-united fractures. Six patients presented with malocclusion at the final follow-up (mean 6.7 months). Of these, one patient was treated with orthognathic surgery, three patients underwent prosthodontic rehabilitation and dental equilibration, and the remaining two patients accepted mild post-treatment malocclusion without any further adjustments. The malocclusion in these patients was judged to be mild.

Group 2: 34 patients were treated solely with dental splints, arch bars, or eyelets along with guiding elastics (Table 3). Seventeen patients were prescribed antibiotics (7–10 days) due to concomitant fractures in the tooth-bearing area which did not require surgical intervention. One patient with an angle fracture developed an infection which was mended with antibiotics. Twenty teeth were involved in the line of fracture, but none required removal. Seven patients were presented with malocclusion at their final follow-up (mean 5.4 months). One patient required orthognathic surgery; four were treated with prosthodontic rehabilitation or dental equilibration, and two accepted the minor malocclusion as it was without further modification.

Group 3: 52 patients did not require any treatment at all; 21 teeth were involved in the line of fracture, and none were removed; 15 patients were prescribed antibiotics (7–10 days) due to fractures in tooth-bearing areas. No infections were reported. Two patients presented with minor malocclusion at the final follow up visit (mean 4 months) but did not express desire for any corrective procedure.

In summary, 19 patients (13.4%) throughout all 3 groups required additional unintended surgical procedures due to complications, such as plate removal, hardware replacement, or orthognathic surgery.

Fifteen patients (10.6%) throughout all groups developed malocclusion after treatment, of whom two required orthognathic surgery to restore normal occlusion (Table 3).

Additional sequelae reported across all groups included objective hypomobility according to our unit’s protocol (MIO < 35 mm), impaired laterotrusion and temporomandibular joint crepitus.

## 4. Discussion

Mandibular fractures represent approximately 36–70% of all facial fractures, with primary treatment goals focused on restoring proper occlusion and promoting fracture healing (7). This study aimed to assess the incidence, types, and distribution of postoperative complications across different fracture types and treatment methods. While open reduction and internal fixation (ORIF) has reported complication rates up to 58%, with infection being the most common, it is associated with faster healing, improved functional recovery, and greater postoperative stability (6).

The role of antibiotics in managing mandibular fractures remains a subject of ongoing debate with considerable variation in postoperative antibiotic protocols. While preoperative antibiotics have been shown to significantly reduce the risk of postoperative infection, evidence supporting the routine use of postoperative antibiotics in uncomplicated cases remains limited [5,6]. Infection rates following ORIF of mandibular fractures can range from 1% to 32% [7,8,9]. In our cohort, 55 patients underwent ORIF, with all patients receiving prophylactic antibiotics for 7 to 10 days. Postoperative infection developed among 23 patients (41.8%) and 52% of those required additional intervention including removal of the infected hardware. Hence, our findings did not support the routine use of postoperative antibiotics as an effective measure to reduce infection rates in the treatment of mandibular fractures. However, without a comparator control group, the effect cannot be reliably assessed. In a systematic review from 2012, Kyzas et al. concluded that the available evidence was insufficient to support the routine use of prophylactic antibiotics in the treatment of mandibular fractures. Furthermore, the review failed to establish a standard protocol or clear criteria for antibiotic administration [10]. Additionally, another systematic review supported the importance of perioperative antibiotics, particularly in cases involving mucosal incisions. While the findings did not support the use of postoperative antibiotics, 64.7% of practitioners included in the review still administered postoperative antibiotics for an average duration of 4.6 days [11]. The generous administration of antibiotics despite the inadequate evidence for their efficacy is similar to our clinic’s standard protocol, where all patients are routinely prescribed antibiotics postoperatively. Despite this, the infection rates in our study (41.8%) were higher compared to outcomes reported in the literature. Gutta et al. conducted a retrospective five-year study analyzing outcomes of mandibular fracture treatment and reported an infection rate of roughly 15%. Furthermore, their findings indicated no correlation between antibiotic use—whether administered preoperatively or postoperatively—and the occurrence of postoperative infections [12].

In a study by a study conducted by Canas et al., it was demonstrated that victims of assault (VOA) who sustain facial fractures are at increased risk for complications—such as infection—due to unfavorable social determinants of health (SDH). There was a higher incidence of infection in open mandibular and maxillary fractures among male VOA patients compared to non-VOA individuals [13]. In our study, 64% of the patients were male, and 22% of the fractures were assault related. These individuals are often presented with poorer SDH and lower compliance with postoperative care, both of which are significant contributing factors to the increased risk of complications and infection. These factors can directly impact their ability to adhere to postoperative care regimens, including medication compliance, wound care, and follow-up appointments. Poor compliance, in turn, contributes significantly to the increased risk of postoperative complications, including infection and may have played a role in the infection rate observed in our cohort. Additionally, the relatively small sample size may have amplified percentage variations. Variations in hardware selection and placement—often influenced by the type of fracture and the individual surgeon’s clinical judgment—may also contribute to the infection rates observed in this study.

Several other factors such as comorbidities, tobacco use and presence of teeth in the line of fracture may also play a role in developing postoperative infection [5,6]. In 2002, Ellis compared infection rates in mandibular angle fractures with and without teeth present in the fracture line and found no significant difference between the two groups [14]. Similarly, Gutta et al. and James et al. reported that the presence or absence of teeth in the fracture line did not affect the incidence of hardware failure. In our study, a total of 58 teeth were found in fracture line, of which 23 were extracted during surgery due to poor conditions or interference with fracture reduction. Consistent with previous findings, our results showed no difference in postoperative infection rates between the two groups. Due to the retrospective design of this study, the potential confounding variables like smoking, alcohol use, and medical comorbidities were not fully measured.

Another important predictor of postoperative complications and infections is the surgical approach—specifically, whether the incision is trans-cutaneous or trans-mucosal. Odom et al. (2016) reported complication and infection rates of 16.8% and 6.6%, respectively, for trans-oral incisions, while trans-cutaneous approaches were associated with no reported complications or infections [15]. Notably, the combined trans-oral and trans-cutaneous approach had the highest complication and infection rates, at 27% and 21.5%, respectively. Interestingly, 70% of patients in our study who required surgery for fractures involving both intraoral and extraoral incisions developed infections in at least one of the surgical sites. This increased risk is likely due to both bacterial contamination from the IO approach and the fact that combined approaches are typically used in more complex, comminuted fractures, where the severity of the injury itself may contribute to the higher complication rate [15].

Highest overall complication rate—including non-union—is typically observed in the mandibular angle region (19–51.7%), followed by the body of the mandible (3.2–34.4%) [5]. In contrast, the condylar region demonstrates a lower incidence of complications, with rates ranging from 10 to 30% [5,16]. Similarly, in our study, the mandibular angle was the most common site for complications. Of the 23 fractures in this region, 11 developed complications and infection, with 6 requiring additional surgery for plate removal and one necessitating replacement of the hardware.

The most reported reason for plate removal includes infection, wound breakdown, plate exposure or patient’s request [17]. Plate removal rates in the mandible have been reported at approximately 6.9%, with the highest incidence occurring in the mandibular angle and body regions [17]. In our study, plate removal was required in 20 fractures (25.6%), primarily involving the angle (*n* = 6) and body (*n* = 7). The higher proportion observed in our cohort is likely attributable to the relatively small sample size and limited number of fractures included.

It has been reported that the prevalence of inferior alveolar nerve (IAN) damage related to mandibular fractures is 33.7% before surgery and increases to 53.8% after surgical treatment following a 12-month follow-up period [18]. Number of miniplates used, the severity of fracture displacement, and the surgeon’s level of experience significantly influence the prevalence of neurosensory deficiency [19]. In our study, the overall prevalence of IAN injury was 67%, without differing between the anterior and posterior mandible after a mean follow-up of 6.1 months. Only 34% of neurological deficits could be attributed to surgical intervention, after accounting for trauma-induced injuries. The relatively short follow-up period has most likely influenced these findings, as nerve recovery typically requires a longer period of time [20].

In this study, fractures requiring transcutaneous incisions were accessed exclusively via submandibular and retromandibular approaches, with 34.7% of patients exhibiting postoperative motor deficits (21.7% following submandibular and 13% following retromandibular incisions). According to the literature, transient facial nerve dysfunction is expected in 15.3% of cases involving submandibular approaches and 14.4% with retromandibular ones [21]. Permanent impairment is less common, reported in 2.2% and 1.4% of patients, respectively, according to a 2018 meta-analysis by Al-Moraissi et al. [21]. Due to the limited follow-up period of nine months in our study, it was not possible to definitively determine whether the observed nerve injuries were temporary or permanent.

In a retrospective study conducted in 2014, Kommers et al. evaluated the need for corrective orthognathic surgery following maxillofacial trauma over a 42-year period in Amsterdam [22]. Their analysis did not specify the number of fractures operated on annually but identified 42 patients requiring orthognathic surgery to correct malocclusion—approximately one case per year. These findings align with our study, where only two patients needed orthognathic surgery during the one-year evaluation period.

It has traditionally been believed that mandibular fractures should be treated within 24 to 48 h. However, recent studies have shown that delayed treatment does not increase complication rates, and there are no significant advantages in either immediate or delayed intervention [5,15]. In our study, the average time from injury to surgical intervention was 3 days. No clear correlation was found between time to surgery and the occurrence of complications.

In this study, 40% of patients underwent surgery during on-call hours, with procedures performed by residents under supervision. The experience level of the operating surgeon is a critical factor influencing outcomes following mandibular fracture repair. Surgeries conducted during off-hours in teaching hospitals are often performed under less optimal conditions, which can introduce variability in outcomes. Differences in technical skill, surgical decision-making, and intraoperative judgment—particularly in managing complex fractures requiring advanced expertise—may adversely affect patient recovery. Together with SDH, these systemic factors, including surgeon experience and timing of surgery, likely contributed to the elevated postoperative complication rates observed. These findings underscore the importance of implementing targeted strategies to enhance perioperative support, surgeon training, and supervision to improve patient outcomes.

Study limitations:

The Oral and Maxillofacial Surgery Department at Karolinska University Hospital manages a high number and wide variety of mandibular fractures. The complication rates observed align broadly with those reported in the literature. However, the proportion of complications in our cohort may differ due to a limited sample size, shorter follow-up periods, and possible confounding factors such as patient comorbidities. The retrospective design limited control over data collection and may have introduced selection bias, with some clinical details or patient-reported outcomes possibly incomplete or inconsistently documented. The relatively short follow-up period (mean 6 months) likely underestimates late complications such as chronic infections and overestimates nerve damage. All surgical patients received prophylactic antibiotics, but without a non-treated control group, the isolated effect of antibiotics on infection rates could not be assessed. Factors such as patient compliance, trauma severity, concomitant injuries, and socioeconomic status were not accounted for, potentially influencing healing and complication rates.

Clinical applicability:

This study offers insights into managing mandibular fractures in a teaching hospital setting, emphasizing the influence of fracture type, treatment approach, and surgical timing on outcomes. It highlights common complications like infections and nerve injuries, aiding surgeons and trainees in anticipating risks and improving surgical planning. The findings also underscore the importance of experience and training in optimizing patient care and minimizing postoperative issues.

## 5. Conclusions

The complication rates observed in this study align with previously reported data. Despite variability influenced by surgeon experience and patient factors, the majority of patients achieved favorable outcomes without requiring reoperation or occlusal rehabilitation. The elevated postoperative infection rate identified warrants further investigation under controlled prospective conditions.

## Figures and Tables

**Table 1:** Demographic Information of Patients with Mandibular Fractures in 2022. Abbreviations: ADHD = attention deficit hyperactivity disorder.

	No	%
Sex		
- Male	91	64.50%
- Female	50	35.50%
Age		
- 12–18	15	10.6%
- 19–65	103	73%
- 66–100	23	16.4%
Fracture		
- Subcondylar	65	29.2%
- Condylar head	53	23.8%
- Symphyseal	47	21.1%
- Angle	32	14.30%
- Body	23	10.30%
- Coronoid	3	1.30%
Fractures/Patient		
- Single	73	51.70%
- Double	54	38.30%
- Triple	14	10%
Cause of Fracture		
- Accidental falls	40	28.40%
- Assault	31	22%
- Bicycle/motor accidents	25	17.80%
- Syncope	18	12.70%
- Other	27	19.10%
Comorbidities		
- Cardiovascular disease	18	12.80%
- Diabetes	16	11.30%
- Smoker	5	3.50%
- Lung disease	4	3%
- Drug/Alcohol abuse	3	2%
- Epilepsy	3	2%
- Cancer	2	1.40%
- Other (e.g., Crohn’s, Bechterew, ADHD)	12	8.50%

**Table 2:** Details of included patients. Group 1 = Surgical treatment. Group 2 = Closed treatment. Group 3 = No treatment. Abbreviations: p = patients; f = fractures.

	Group 1	Group 2	Group 3	Total
Patients (*n*)	55	34	52 *	141
Fractures (*n*)	99 (78 operated)	52	72	223
- *Symphyseal*	27	13	7	
- *Angle*	23	1	8	
- *Subcondylar*	15	26	14	
- *Body*	13	4	5	
- *Condylar head*	0	8	37	
- *Coronoid*	2	0	1	
Tooth in fracture line	58 (Removed: 23)	20	21	
Infection	23 p, 27 f	1 p, 1 f	0	
*-* Healed with antibiotics	11 p, 12 f	1 p, 1 f	-	
Follow up visits (Mean)	7	6	3	
Days from trauma to treatment (Mean)	3	3	4	
Malocclusion	6 p, 11 f	7 p, 12 f	2 p, 3 f	15 p
- Follow up months (Mean)	6.7	5.4	4	
Corrective procedures				
*-* Dental equilibration	2	3	0	5
*-* Orthognatic surgery	1	1	0	2
*-* Prosthodontics	1	1	0	2
*-* No treatment	2	2	2	6

Note: * Two patients were edentulous, and eight patients did not attend continued follow-up appointments; therefore, their occlusion was not evaluated and is not included in this table.

**Table 3:** Details of infection, plate removal and other complications in group 1. Abbreviations: SS = surgical sites. RCT = root canal treatment. TMJ = temporomandibular joint.

Fracture Location	Symphyseal	Angular	Subcondylar	Condylar Head	Body	Coronoid	Total
Surgical treatment	27	23	15	0	13	0	78
Non surgical treatment	20	9	50	53	10	3	145
Infection (SS)	9	9	3		6		27
Lysis/discomfort	1	2	-		2		5
Plate removal	7	6	-		7		20
New plating	4	1	-		2		7
Salivary fistula	-	-	3		-		3
Aneurysm	-	-	-		1		1
RCT (teeth)	-	2	-		-		2
TMJ disorder	-	-	1		-		1

## Data Availability

Data availability is restricted due to privacy and ethical considerations.

## Abbreviations

The following abbreviations are used in this manuscript:

ORIF	Open reduction and internal fixation
IMF	Intermaxillary fixation
IAN	Inferior alveolar nerve
FN	Facial nerve
SSI	Surgical site infection
RCT	Root canal treatment
TMJ	Temporomandibular joint
VOA	Victims of assault
SDH	Social determinants of health
